# Effects of temperature and storage duration on quality of an insect artificial diet

**DOI:** 10.3389/finsc.2024.1475411

**Published:** 2024-09-18

**Authors:** Khanh-Van Ho, Bruce E. Hibbard, Michael G. Vella, Kent S. Shelby, Man P. Huynh

**Affiliations:** ^1^ Metabolomics Center, University of Missouri, Columbia, MO, United States; ^2^ Department of Biochemistry, University of Missouri, Columbia, MO, United States; ^3^ Plant Genetics Research Unit, United States Department of Agriculture (USDA)-Agricultural Research Service, Columbia, MO, United States; ^4^ Frontier Agricultural Sciences, Newark, DE, United States; ^5^ Biological Control of Insects Research Laboratory, United States Department of Agriculture (USDA)-Agricultural Research Service, Columbia, MO, United States; ^6^ Division of Plant Science and Technology, University of Missouri, Columbia, MO, United States

**Keywords:** diet stability, completed diets, insect rearing, insect assays, *Diabrotica* spp., corn rootworms

## Abstract

Artificial diets are widely used to produce insects for research and education programs. Completed diets, in which the diets are fully made from individual ingredients and ready to use, often have high water activity, making them vulnerable to degradation. Proper storage is critical to maintaining diet quality, yet the storage conditions are not well investigated. In this study, we characterized the effects of storage conditions (temperatures and storage duration) on the quality of a diet capable of rearing both specialist and generalist insect species. The completed diet, produced by both private industry and a USDA-Agricultural Research Service laboratory, was exposed to varying temperatures during a 24-hour transit over 1600 km. After transit, it was stored at 4°C for a total storage period of 28 days. In a separate experiment, the completed diet was stored immediately after diet production at five fixed temperatures (-20, 4, 22, 25, and 33°C) for up to 28 days. For both experiments, at 5 intervals after storage (1, 7, 14, 21, and 28 days), diet quality was accessed by life history parameters (survival, molting, and weight) of western corn rootworm (*Diabrotica virgifera virgifera* LeConte) larvae, the most serious maize pest in the United States. Our results showed that exposure to varying temperatures between -2°C and 27°C for 24 hours had no significant impact on diet quality. However, extended storage (beyond 24 hours) at any of the fixed temperatures negatively affected diet quality. Insects reared on diets stored for over 24 hours at fixed temperatures ranging from -20°C to 33°C had significant declines in performance. Among the tested temperatures, -20°C and 4°C were found to be the most effective for preserving diet quality. At these low temperatures, there were no significant changes in insect weight and survival for diets stored within 21 and 28 days, respectively, though molting was significantly reduced within 7 days of storage. These findings provide the base of information on the storage conditions for completed diets, supporting the production of healthy insects.

## Introduction

Agricultural insect pests present one of the most significant threats to global food security. Annual economic losses as a direct result of these arthropod pests are estimated at $470 billion worldwide annually (1). With rising temperatures, crop production losses to agricultural pests are projected to significantly increase by 10% to 25% per degree of global mean surface warming (2). Mitigating this ever-present threat requires a concerted effort by both public and private institutions to routinely develop new management methods for insect pest management. Entomological research requires conducting experiments with insect specimens, which relies heavily on the ability to produce insects using artificial diets (3). Rearing insects on artificial diets is simpler, more controlled and convenient in many ways compared to rearing on natural host plants (4, 5).

Insect artificial diets are generally found in one of two forms such as mixtures of individual ingredients or completed diets. According to Cohen (5), completed diets are those that have been fully synthesized from their individual ingredients and are ready to use, whereas the mixtures of individual ingredients typically refer to the combinations of ingredients that do not include water and have not undergone any production processes. While the individual ingredients are usually stable, completed diets often have a shorter shelf-life. Most dry ingredients (moisture contents of <10%) preserve their nutritional value and palatability for months under low temperatures (e.g., <0°C) (5). Completed diets typically consist of 70% - 95% water (w/w) (6) and possess high water activity, which promotes oxidation, nutrient degradation, and microbial attack (7). Even under cold conditions, degradation of completed diets can continue to take place (5). Inappropriate storage of completed diets can result in reductions in nutrient quality and development of toxins from microbial infestations, causing detrimental effects on the growth and development of insects fed on these diets. Cohen and Crittenden (8) reported the destruction of nutrients (i.e., ascorbate) in three completed diets can occur within 48 hours in a refrigerator or in rearing room conditions. Despite these findings, the stability of completed diets under different temperature and storage duration conditions has not been characterized.

Artificial diets have been a key component in research programs to monitor resistance development and develop new insecticidal toxins and other control agents (e.g., entomopathogenic nematodes) to manage insect pests of maize (9–16). The western corn rootworm, *Diabrotica virgifera virgifera* LeConte (Coleoptera: Chrysomelidae), is the most important pest of maize in the United States and some parts of Europe (17). This insect species is one of the world’s most expensive pests to control, costing up to $2 billion annually in losses and control costs to U.S. maize growers (18).

In this study, a recently developed diet capable of supporting both generalist and specialist insect species in the corn rootworm complex (*Diabrotica* spp.) including the western corn rootworm was selected (19). The goal of the present study is to determine the effects of temperature (varying or fixed) and storage duration on the quality of a completed diet using the universal diet. The completed diet was prepared by both a private industry (Frontier Agricultural Sciences, Newark, Delaware) and a USDA-Agricultural Research Service laboratory (USDA-ARS Plant Genetics Research Unit (PGRU), Columbia, MO). The completed diet was exposed to different varying temperatures during transit for approximately 24 hours from Frontier Agricultural Sciences to the USDA-ARS PGRU (distance ~1600 km) followed by storage at 4°C for a total storage period of 28 days. In a separate experiment, the completed diet was stored immediately after diet production at five fixed temperatures (-20, 4, 22, 25, and 33°C) for up to 28 days. The quality of the completed diet at intervals after storage of 1, 7, 14, 21, and 28 days was evaluated by life history parameters (survival, molting, and weight) of the western corn rootworm larvae reared on the diets.

## Materials and methods

### Artificial diet

A universal diet for corn rootworms (*Diabrotica* spp.) capable of rearing both specialist and generalist insect species including the western corn rootworm, the northern corn rootworm (*D. barberi* Smith & Lawrence), and the southern corn rootworm (*D. undecimpunctata howardi* Barber) was utilized (19). This artificial diet also supports the development of the Mexican corn rootworm (*D. virgifera zeae* Krysan & Smith) (Huynh et al., unpublished). The universal diet, detailed in Huynh, Hibbard, Vella, Lapointe, Niedz, Shelby and Coudron (20), is comprised of 15 ingredients (e.g., wheat germ, whole egg powder, cellulose, vitamin mix, salt mix, preservatives, water) that are commonly used in many insect diets (5). A single batch of its dry mixture, prepared by Frontier Agricultural Sciences, was used for all experiments.

### Insects

Adults of a non-diapausing strain of the western corn rootworm were maintained at the USDA-ARS PGRU as described previously (21). This strain, originally established from eggs purchased from Crop Characteristics (Farmington, MN), has been maintained on a non-transgenic maize line (Viking 60-01N, Albert Lea Seed, Albert Lea, MN) for multiple generations (22). The adult beetles were reared on an artificial diet (23) and water in 30 × 30 × 30 cm cages (BugDorm^®^, BioQuip Products Inc., Rancho Dominguez, CA). Petri dishes (9-cm, Fisher Scientific, Pittsburgh, PA) containing 80-mesh sieved soil as an ovipositional medium were placed in the rearing cages to collect eggs (24). After the eggs were collected, the egg dishes were incubated at 25 ± 1°C in darkness for prompt use or were stored at 8 ± 1°C for later use in an incubator (Percival, Perry, IA) (10).

### Egg treatment

Before conducting the bioassays, the egg dishes were incubated at 25 ± 1°C in darkness in a Percival incubator. Once several eggs had hatched, the eggs were surface-sterilized according to previously described procedures (25, 26) with modifications. Briefly, the mixtures of eggs and soil were rinsed through a 250-µm, 60-mesh sieve (Hogentogler & Co. Inc., Columbia, MD) to remove soil. The eggs retained in the sieve were collected into a 50 mL beaker using a stream of tap water. The eggs were then rinsed with tap water to remove neonates and hatched egg cases that floated to the water’s surface. Next, the eggs were treated with undiluted Lysol (10 ml, Clean & Fresh Multi-Surface Cleaner, Reckitt Benckiser, Parsippany, NJ) for 3 min, followed by a triple rinse with purified water to remove the Lysol solution. The eggs were then rinsed with 10% formaldehyde solution (10 ml, HT501128, Sigma Aldrich, St. Louis, MO). After removing the formaldehyde solution, the eggs were triple rinsed with purified water and were dispensed to a coffee filter paper (Pure Brew, Rockline Industries, Sheboygan, WI). The filter paper was then placed inside a container with a lid (16-oz, LG8RB-0090 & DM16R-0090, Solo Cup Company, Lake Forest, IL) and incubated at 25 ± 1°C in darkness in the Percival incubator. Larvae that hatched within < 24 h were used for the insect bioassays.

The eggs can be surface-sterilized to obtain larvae for multiple days. After the initial egg treatment, in the following days, the eggs were washed with 10% formaldehyde solution for 3 min, followed by a triple rinse with purified water. The eggs were then dispended to a new filter paper in a 16-oz container and were incubated as described above to collect the newly hatched larvae (< 24 h old).

### Effects of varying temperatures during transit and storage duration on diet quality

The artificial diet was prepared in 96-well plates by Frontier Agricultural Sciences using a procedure described previously (20) with modifications. Briefly, agar was added to purified water and the agar solution was boiled using a microwave until the agar was completely melted. The dry mixture was poured into the agar solution when it cooled to approximately 65°C. The plates (96-well plate, 3370, Corning Inc., Corning, NY) were then filled with diet solutions (200 µl per well) using a repeater pipette (HandyStep^®^ S, Brandtech, Essex, CT). The diet plates were sealed with an adhesive sealer (AB-0558, Thermo Scientific™, Waltham, MA). All fills occurred in a laminar flow hood (1800 Series, Thermo Scientific™) to prevent incidental contamination. All filled diet plates were prepared at the same time.

The completed diet plates were allowed to be exposed to varying temperature conditions experienced during transit for 24 hours from Frontier Agricultural Sciences to the USDA-ARS PGRU. The filled diet plates were packed in separate Styrofoam shipping boxes filled with either hot packs, cool packs, ice packs, or dry ice to mimic varying potential environmental conditions experienced during transit. Five different temperatures ranging from 0°C to 30°C were targeted. During the transit, the box temperatures were monitored at one minute intervals using a data logger (UX100-003, Onset, Bourne, MA) placed inside each box. Upon receipt, the diet plates were immediately stored at 4°C for up to 28 days. At 5 durations after storage (1, 7, 14, 21, and 28 days), the quality of the prepared diet was evaluated by life history parameters (survival, molting, and weight) of the western corn rootworm larvae reared on diets.

### Effects of fixed temperatures and storage duration on diet quality

The completed diet was prepared in 96-well plates (3370, Corning Inc.) at the USDA-ARS PGRU as described previously (20) using the same dry diet batch as used for the transit experiment. After the completed diet plates were made, the diet plates were immediately stored at 5 different fixed temperatures (-20, 4, 22, 25, and 33°C) for 5 durations (1, 7, 14, 21, and 28 days). Diet assays were conducted to evaluate the effects of these fixed temperatures on diet quality immediately after each predetermined duration. The quality of the artificial diet was assessed via the evaluation of life history parameters (weight, molting, and survival) of the western corn rootworm larvae reared on the diets.

### Insect diet bioassays

The bioassays were conducted as described previously (27). All materials used in the diet assays were surface-treated via exposure to UV light for 10 min in a biological cabinet (SG403, SterilGARD^®^ III Advance cabinet, Sanford, ME). Each treatment was a completed diet exposed to different temperatures and time intervals of storage. Each replicate included the artificial diet pipetted to a 12-well row of the 96-well plate. Each treatment was replicated 6 times in different diet plates. Each well was infested with one western corn rootworm neonate (< 24 h old) using a fine paintbrush. A sealing film (TSS-RTQ-100, Excel Scientific, Inc., Victorville, CA) was used to cover the plate. For ventilation, a hole was made in the sealing film over each well using a number zero insect pin. The plates were kept in a Percival incubator at 25°C in darkness for 7 days. Larval molting, survival, and evidence of contamination (fungi and bacteria) were recorded daily, whereas larval weight was measured at the end of the experiments. During the experiment, larvae were typically found on the surface of the diet. Larval molting was recorded by observing the presence of the old cuticles that the larvae shed during the molting process on the diet’s surface, while larvae were considered dead if they did not exhibit coordinated movement after being touched with the number zero insect pin. For larval dry weight, all live larvae in each treatment were pooled per replicate (12 possible) into 95% ethanol, dried in an oven (Binder GmbH, Tuttlingen, Germany) at 55°C for 48 h, and weighed using a micro balance (MSU6.6S-000-DM, Sartorius Lab Instruments GmbH & Co. KG, Goettingen, Germany).

### Data analyses

To determine survival and molting rates, the number of live larvae and successful larval molts from 1st to 2nd instar per replicate were divided by the initial number of larvae and multiplied by 100 to obtain percentages. The weight per larva (in mg) was calculated by dividing the dry weight by the number of live larvae per replicate. If all larvae for a replicate were dead, the larval weight for this replicate was recorded as 0.

The experiments were designed as a 2-factor factorial design (temperature × time). The life history data, including percent survival, weight, and percent molt to second instar, were analyzed separately using analysis of variance (ANOVA) in generalized linear mixed models (PROC GLIMMIX) in SAS 9.4 (SAS Institute, Cary, NC). Temperatures and time exposure were the fixed effects and replication was the random variable. Differences between treatments were determined using Fisher’s least significant difference (LSD) at p < 0.05. The percent variables (survival and molt) were arcsine square-root transformed and the numeric variable (weight) was square-root transformed prior to the analysis to meet the assumption of normality and homoscedasticity. The untransformed data were presented as MEAN ± SEM.

## Results

### Effects of varying temperatures during transit and storage duration on diet quality

**Figure 1:**
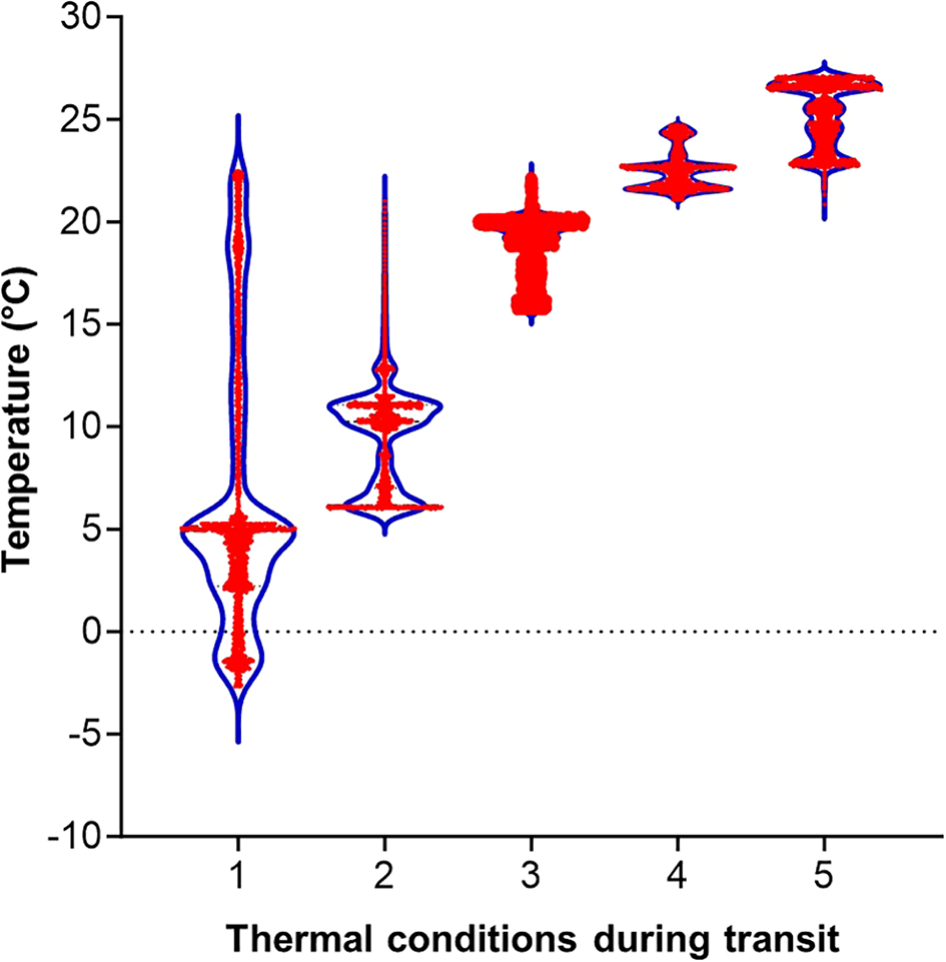
Temperature exposure to diet plates during the transit for approximately 24 hours. Violin shapes show distributions of temperatures, measured at one minute intervals, for 24 hours in each treatment. Red dots are data points.

The diet plates arrived at the USDA-ARS PGRU (Columbia, MO) after departing approximately 24 hours from Frontier Agricultural Sciences (Newark, DE). During the transit, the complete diet was exposed to 5 different averaged temperatures of 6.6 ± 6.7°C, 9.7 ± 2.7°C, 18.8 ± 1.5°C, 22.5 ± 1.0°C, and 25.2°C ± 1.6°C with major temperature exposure from -2°C to 27°C (**Table 1**; **Figure 1**). Upon receipt, all the diet plates were visually examined, and they were presented in good physical condition (i.e., gel matrix intact, no constituent separation, no excess moisture, no desiccation) and were stored at 4°C for up to 28 days.

**Table 1:** Temperature exposure of diet plates during the transit for approximately 24 hours.

Thermal conditions during transit	Averaged temperature exposure	Major temperature exposure
1	6.6 ± 6.7°C	-2°C - 6°C
2	9.7 ± 2.7°C	6°C - 12°C
3	18.8 ± 1.5°C	15°C - 19°C
4	22.5 ± 1.0°C	21°C - 24°C
5	25.2 ± 1.6°C	23°C - 27°C

**Figure 2:**
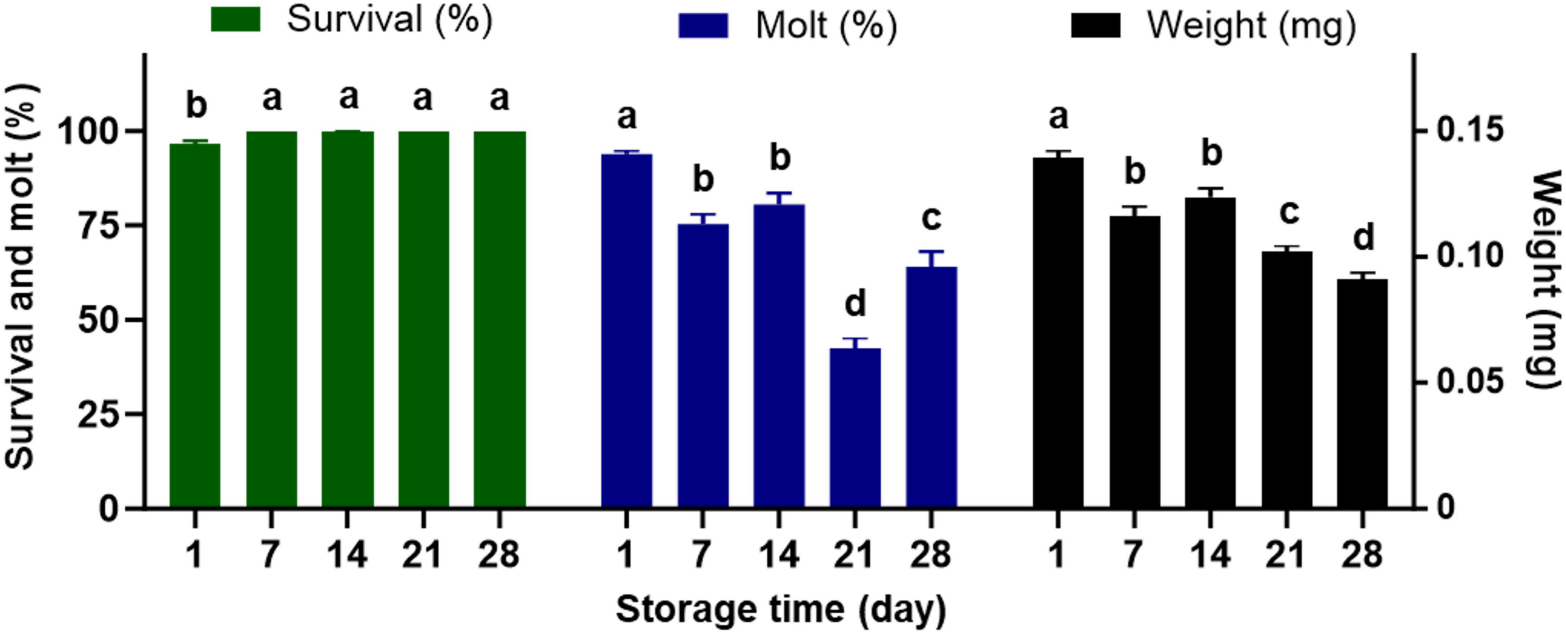
Percent survival, dry weight, and percent successful completion of molt to 2nd instar for western corn rootworm larvae reared on an artificial diet for 7 days. During the transit, the completed diet was exposed to varying temperatures ranging from -2°C to 27°C for approximately 24 hours. Upon receipt, the completed diet was stored at 4°C for 5 time points (1, 7, 14, 21, and 28 days). Bars with different letters are significantly different (P < 0.05). Mean ± SEM. Untransformed data were presented, while analyses were performed with square-root transformed or arcsine square-root transformed data.

Exposure to varying temperatures from -2°C to 27°C for approximately 24 hours during the transit had no significant impacts on the quality of the completed diet assessed by life history parameters measured (survival, molt, and weight) (**Table 2**). However, the duration of storage at 4°C after shipping had significantly detrimental effects on the quality of the artificial diet for feeding the western corn rootworm larvae (**Table 2**). With respect to time of storage with all varying temperatures combined (**Figure 2**), significant reductions in larval molting and weight were observed for the completed diets stored within 7 days. There was no significant difference in larval weight and molting between 7 and 14 days after storage, whereas the lowest larval molting and weight were found at 21 and 28 days of storage, respectively. Larval survival remained above 95% at all time points evaluated, indicating that diet storage for up to 28 days did not adversely affect survival (**Figure 2**). The interactions between temperature × time were not significantly different for any of the life history parameters (**Table 2**).

**Table 2:** Effects of temperature (varying) and storage duration on quality of an artificial diet.

Parameter	Effect	df	F	P
Survival	Temperature	4, 120	0.30	0.8804
	Time	4, 120	9.42	<0.0001
	Temperature × Time	16, 120	0.33	0.9934
Molting	Temperature	4, 120	2.33	0.0599
	Time	4, 120	44.18	<0.0001
	Temperature × Time	16, 120	1.42	0.1420
Weight	Temperature	4, 120	2.27	0.0660
	Time	4, 120	34.19	<0.0001
	Temperature × Time	16, 120	1.57	0.0869

The diet quality was assessed by life history parameters (survival, molting, and weight) of western corn rootworm larvae reared on the artificial diet.

### Effects of fixed temperatures and storage duration on diet quality

**Figure 3:**
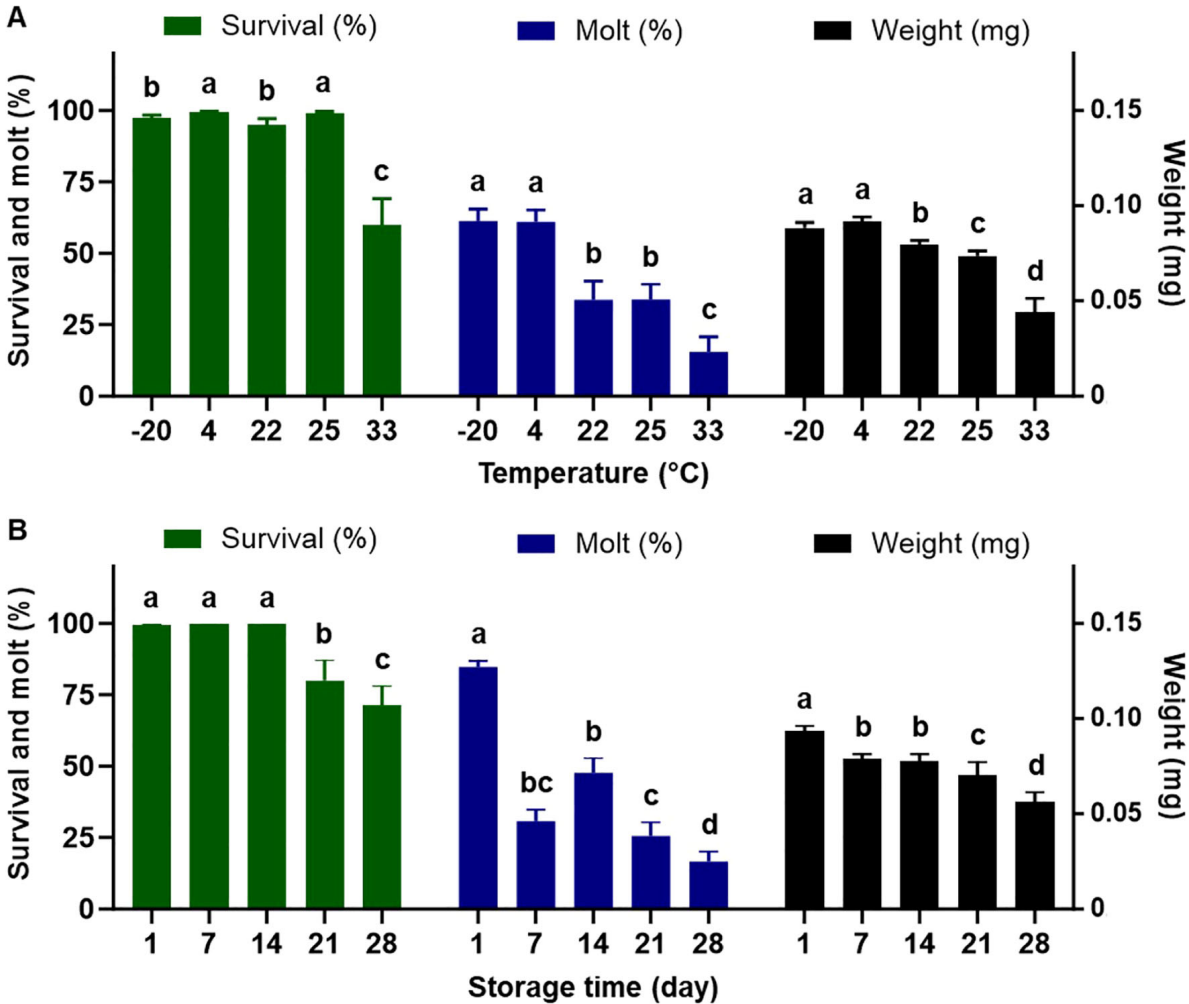
Percent survival, dry weight, and percent successful completion of molt to 2nd instar for western corn rootworm larvae reared on an artificial diet for 7 days. **(A)**: storage duration with all temperatures combined, **(B)** temperatures with all the storage duration combined. Bars with different letters are significantly different (P < 0.05). Mean ± SEM. Untransformed data were presented, while analyses were performed with square-root transformed or arcsine square-root transformed data.

The fixed temperatures, time of storage, and their interactions significantly impacted the quality of the completed diet for rearing the western corn rootworm larvae (**Table 3**). With respect to the temperatures with all the time of storage combined (**Figure 3A**), averaged larval survival was over 95% for temperatures from -20°C to 25°C. In contrast, survival declined significantly to 60% for the highest temperature of 33°C. Temperatures of -20°C and 4°C had the highest larval weight and molting rates, followed by temperatures of 22°C and 25°C. The temperature of 33°C had the lowest rates for all examined parameters (weight, molting, and survival). Regarding storage duration with all temperatures combined (**Figure 3B**), the completed diet stored for 7 days had a significantly negative impact on larval molting and weight. A significant reduction in larval survival was found starting after 21 days of storage.

**Table 3:** Effects of temperature (fixed) and storage duration on quality of an artificial diet.

Parameter	Effect	df	F	P
Survival	Temperature	4, 120	332.24	<0.0001
	Time	4, 120	272.67	<0.0001
	Temperature × Time	16, 120	134.14	<0.0001
Molting	Temperature	4, 120	72.94	<0.0001
	Time	4, 120	114.12	<0.0001
	Temperature × Time	16, 120	5.00	<0.0001
Weight	Temperature	4, 120	264.21	<0.0001
	Time	4, 120	106.51	<0.0001
	Temperature × Time	16, 120	66.39	<0.0001

The diet quality was assessed by life history parameters (survival, molting, and weight) of western corn rootworm larvae reared on the artificial diet.

**Figure 4:**
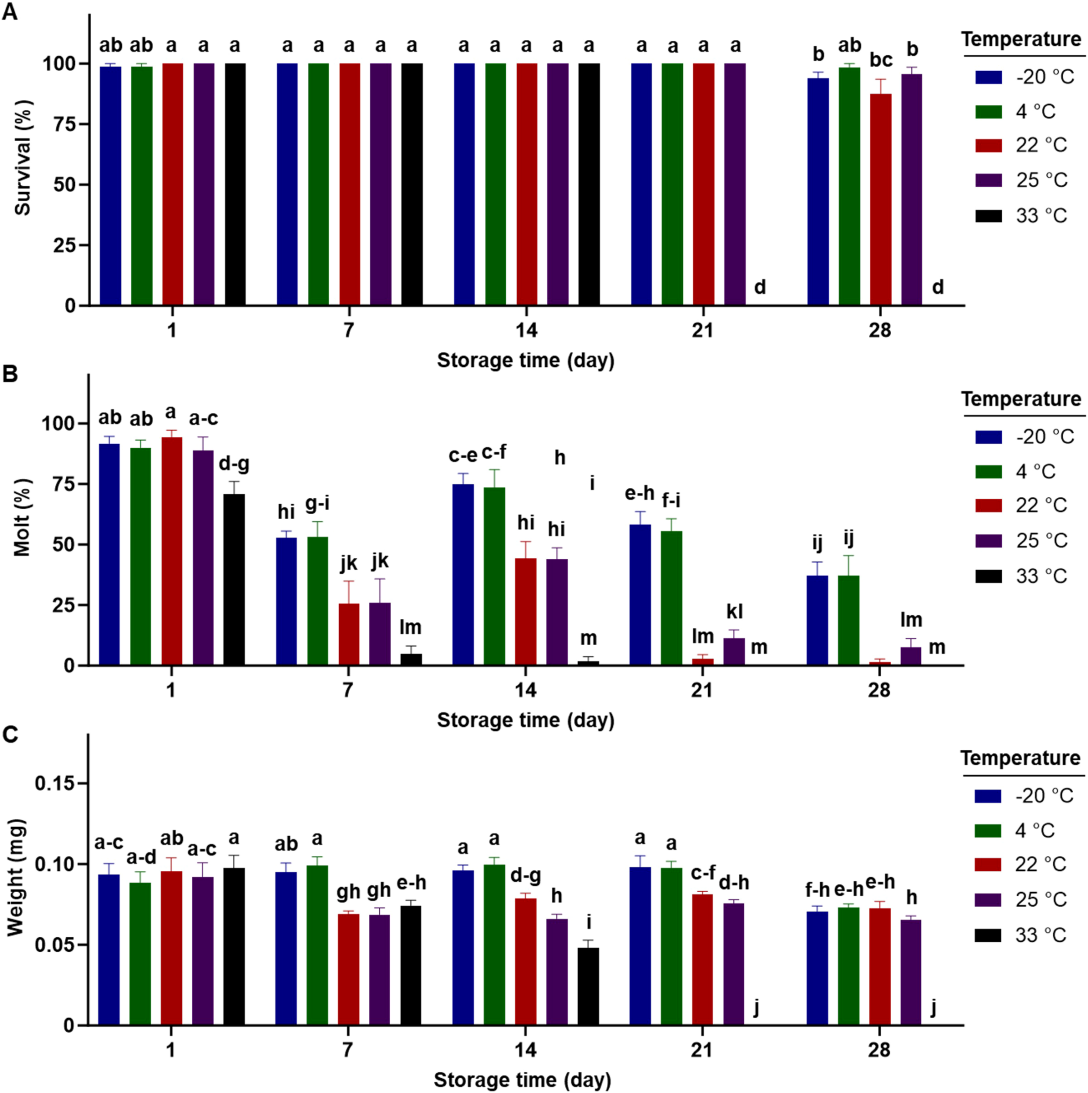
Percent survival **(A)**, dry weight **(B)**, percent successful completion of molt to 2nd instar **(C)** for western corn rootworm larvae reared on an artificial diet for 7 days. Bars with different letters are significantly different (P < 0.05). Mean ± SEM. Untransformed data were presented, while analyses were performed with square-root transformed or arcsine square-root transformed data.

**Figure 4** reveals the interactions between temperature and storage duration for each examined parameter. Over a 14-day storage period, there was no significant difference in larval survival on the completed diet stored at any temperature (**Figure 4A**). However, significant reductions in survival were observed primarily at the highest temperature (33°C), with no survival found after 21 days of storage (**Figure 4A**). Significant declines in molting began at all temperatures after 7 days of storage, except for the highest temperature (33°C) where reductions occurred after just 1 day of storage (**Figure 4B**). After 7 days of storage, diets stored at -20°C and 4°C supported greater larval molting compared to those stored at higher temperatures. Larval weight at the lowest two temperatures remained unaffected within 21 days of storage (**Figure 4**).

### Contamination

No evidence of contamination (bacteria or fungi) was observed during all experiments, except for the temperature storage at 33°C. After 21 days of storage at 33°C, the completed diet was mostly contaminated with bacteria. This contamination contributed to 100% mortality of the insects fed on these diets (**Figure 4A**).

## Discussion

Artificial diets are critical to many programs as they are used to rear insects for research across various entomological fields (e.g., insect management in agriculture, development of nutrient sources for human consumption or animal feed) and other biological sciences (3, 5). The success of these programs hinges on the health of the insects, which is directly affected by the quality of the diets (28). Although insects are frequently reared on completed diets requiring proper storage to maintain their quality, there is limited information available on the storage conditions of completed diets. In this study, by characterizing temperature and time storage conditions, we determined the quality of a completed diet after storage at different varying and fixed temperatures from -20°C to 33°C for up to 28 days.

Our results indicated exposure to varying temperatures from -2°C to 27°C for less than 24 hours during transit had no significant effect on the quality of the completed diet (**Table 1**). However, longer storage time (>24 hours) at different temperatures caused negative impacts on diet quality. In fact, the performance of the western corn rootworm larvae fed on the completed diet stored over 24 hours at fixed temperatures ranging from -20°C to 33°C was significantly reduced (**Figures 2**, **3**). Among the temperatures examined, low temperatures (-20°C and 4°C) proved to be the most effective for preserving diet quality. Insects fed on diets stored at these low temperatures exhibited higher weight and better molting rates compared to those reared on diet stored at higher temperatures (22°C, 25°C, and 33°C) (**Figures 2**–**4**). The observed reductions in insect performance are likely due to nutrient degradation in the completed diet, which can occur quickly after storage. Cohen and Crittenden (8) reported significant declines in ascorbic acid in these completed diets for plant bugs, lepidopteran larvae, and green lacewings within 48 hours of storage in a refrigerator at 4°C or in rearing room conditions at 27°C. Notably, the completed diet used in this study contains 80% water (20). Insect diets that often have over 75% water and thereby possess high water activity (>95%) which facilitates various forms of degradation (e.g., oxidative deterioration), attributing to the instability of completed diets (5, 6).

Among the life history parameters examined, it is clear that molting was the parameter that was the most affected (**Figure 4**). At low temperatures (-20°C or 4°C), significant reductions in larval molting were seen when the insects fed on completed diets stored within 7 days, whereas declines in larval weight and survival did not occur within 21 and 28 days, respectively, for diets stored at such low temperatures. Sterols, which are precursors to ecdysteroids (molting hormones) of insects, are often added to insect diets as a vital ingredient. Since insects cannot synthesize sterols, they must obtain them from their diet (29). During storage, lipid oxidation in completed diets might occur rapidly, resulting in declines in the availability of sterols, or possibly other lipids such as fatty acids, which are essential for juvenile hormone production. Cholesterol, a common ingredient in insect diets, provides sterols necessary for insect growth and development. The completed diet used in this study contains 0.0055% cholesterol (20). Increasing the proportion of cholesterol in this diet may mitigate the loss of sterols.

Researchers in insect rearing often encounter challenges with the problem of diet preservation. The standard techniques for preserving human food do not always translate to insect diets. Most completed diets are composed of a complex mixture of several ingredients with high water activity, making them susceptible to various kinds of degradation (5). Even before insects contact the completed diet, interactions among diet components during processing and storage can affect its quality. Moreover, infestation of diets with insects negatively impacts the biochemical integrity of the diet. Insect feeding accelerates oxidative deterioration by the introduction of digestive secretions, increasing exposure to atmospheric oxygen, and contamination of the diet with microbes from their mouthparts and frass (30).

To produce healthy insects on artificial diets, it is essential to maintain diet quality under appropriate conditions while considering economic feasibility. This study adds to the limited number of studies characterizing the effects of storage conditions (temperature and storage duration) on the quality of insect diets. Short-term exposure (less than 24 hours) to varying temperatures between -2°C and 27°C had no adverse effects on diet quality, but prolonged exposure (over 24 hours) at any fixed temperatures from -20°C and 33°C deteriorated the completed diets. The best preservation of diet quality was achieved at -20°C and 4°C, where insect weight and survival remained unaffected within 21 and 28 days of storage, respectively, though molting was reduced within 7 days of storage. This provides essential information on the storage conditions for completed diets, supporting the production of healthy insects.

## Data Availability

The original contributions presented in the study are included in the article/supplementary material. Further inquiries can be directed to the corresponding authors.
